# Harnessing Artificial Intelligence in Pediatric Oncology Diagnosis and Treatment: A Review

**DOI:** 10.3390/cancers17111828

**Published:** 2025-05-30

**Authors:** Mubashir Hassan, Saba Shahzadi, Andrzej Kloczkowski

**Affiliations:** 1 The Steve and Cindy Rasmussen Institute for Genomic Medicine, Nationwide Children’s Hospital, Columbus, OH 43205, USA; mubasher.hassan@nationwidechildrens.org (M.H.); saba.shahzadi@nationwidechildrens.org (S.S.); 2 Department of Pediatrics, The Ohio State University, Columbus, OH 43205, USA; 3 Department of Biomedical Informatics, The Ohio State University, Columbus, OH 43210, USA

**Keywords:** pediatric oncology, artificial intelligence, cancer diagnosis, machine learning, deep learning

## Abstract

Artificial intelligence (AI) is rapidly transforming pediatric oncology by creating new means to improve the accuracy and efficacy of cancer diagnosis and treatment in children. This review critically examines current applications of AI technologies like machine learning (ML) and deep learning (DL) to the main types of pediatric cancers. However, the application of AI to pediatric oncology is prone to certain challenges, including the heterogeneity and rarity of pediatric cancer data, rapid technological development in imaging, and ethical concerns pertaining to data privacy and algorithmic transparency. Collaborative efforts and data-sharing schemes are important to surpass these challenges and facilitate effective training of AI models. This review also points to emerging trends, including AI-based radiomics and proteomics applications, and provides future directions to realize the full potential of AI in pediatric oncology. Finally, AI is a promising paradigm shift toward precision medicine in childhood cancer treatment, which can enhance the survival rates and quality of life for pediatric patients.

## Simple Summary

Artificial intelligence (AI) is increasingly being used in medicine, especially in diagnosing and treating childhood cancers. Pediatric oncology faces unique difficulties due to the rarity and diversity of these cancers, making the use of AI promising for more accurate and efficient detection and treatment. AI analyzes large amounts of data, like medical images and genetic information, faster than doctors can. It helps identify tumors and cancer types and predicts treatment responses, leading to quicker and more tailored diagnoses for children. However, using AI in pediatric cancer has challenges due to limited data on rare cancers in children. This makes training AI systems difficult. Continuous advancements in AI technology and information-sharing between hospitals and research centers are addressing these issues.

## 1. Introduction

Pediatric malignancies are a major cause of death for children worldwide and throw a sinister shadow over the lives of many children and their families [1]. Despite significant progress in medical research and treatment advances, the fight against pediatric malignancies is still a complex and challenging endeavor [2]. A vast number of children receive cancer diagnoses, including sarcomas, brain tumors, neuroblastomas, and leukemia, which forces them to fight an intangible foe [3,4]. Acute leukemia, which makes up nearly 28% of all pediatric cancer cases, is the most prevalent type of the disease. Acute myeloid leukemia (AML) and acute lymphocytic leukemia (ALL) are the most pervasive forms of cancer in youngsters [5]. Brain and spinal cord tumors rank second in terms of frequency among pediatric cancers, making up approximately 26% of all pediatric cancer cases. Brain and spinal cord tumors can take many distinct forms, and each one has a unique etiology and course of treatment [6]. Because of the severe side effects of radiation and chemotherapy, as well as sometimes delayed diagnosis, treating juvenile tumors encounters different challenges compared to treating adult cancers. It is critical to comprehend all difficulties and circumstances associated with various pediatric malignancies and create innovative treatment strategies [7,8].

Rapid advancements in knowledge and technology have opened new avenues for multi-layered diagnoses in pediatric oncology [9]. Artificial intelligence (AI) techniques can quickly process vast volumes of original data to solve intricate problems with high accuracy [10]. Therefore, with AI approaches, doctors can potentially increase the effectiveness of their diagnosis, improving the subsequent individualized treatment and surveillance [11,12]. AI systems can gather data, recognize underlying patterns, reach predetermined goals, and make judgments and forecasts regarding actual occurrences. As a primary subset of artificial intelligence (AI), machine learning (ML) denotes a distinct approach from conventional hard-coded software programs that use algorithms to build prediction models dynamically by training on vast volumes of historical data [13,14]. Deep Learning (DL) is a rapidly expanding field of artificial intelligence (AI) that leverages the structure of convolutional neural networks (CNNs) with numerous interconnected layers to provide learnable weights and high efficiency with little pre-processing [15]. DL-CNNs, which are made up of several stacked CNN layers, are superior to earlier ML algorithms in terms of accuracy, speed, and vendor independence [9].

Machine learning (ML), the core domain of artificial intelligence, allows computers to learn from vast data and improve their decision-making capabilities without being explicitly coded [16]. In oncology, the research and treatment of pediatric cancer, ML has been recognized as a central technology to enhance diagnosis, classify cancer in the best way, and inform customized treatment processes [17]. Because cancer is a public health issue due to its high incidence, death, and biological diversity, the utilization of machine learning offers potential avenues to improve early detection and prognosis [18]. ML algorithms applied in oncology aim to complement traditional clinical methodologies and enable better, more precise, and data-driven healthcare practices [19,20]. In cancer research, AI techniques have provided beneficial support for clinical management, including cancer segmentation, susceptibility, and classification, which are crucial components for early detection and prognosis management [21,22]. Despite the current AI advancements, the sustainable development of AI tools in health care relies on the availability of large datasets with strict quality control. Different AI-based algorithms and approaches can be used can be used to assist in the diagnosis and management of pediatric cancers [23].

## 2. AI-Based Application for Pediatric Diagnosis and Treatment

**Figure 1:**
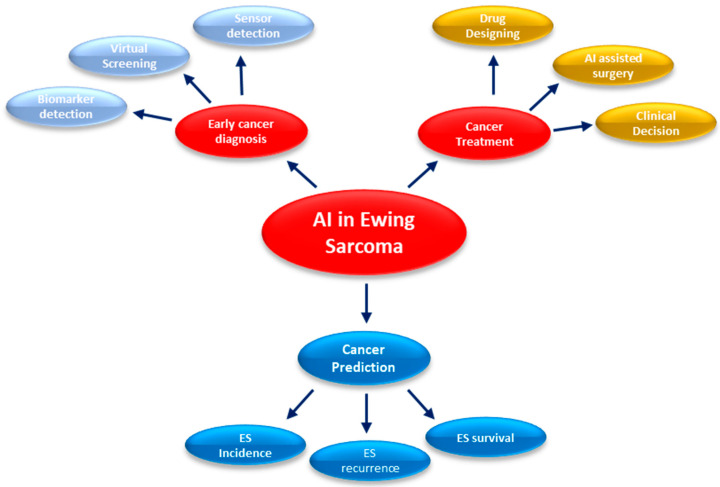
Potential uses of AI in Ewing Sarcoma management.

To illustrate various applications of AI-based methods in pediatric oncology, we will focus on Ewing Sarcoma (ES), a rare type of bone cancer that primarily affects kids and youngsters. AI-based methods can be used in cancer prediction, early cancer diagnosis, and cancer treatment, as shown in Figure 1.

### 2.1. Early Detection and Diagnosis for ES

#### 2.1.1. Image Analysis

AI algorithms can analyze medical images, such as computed tomography (CT) scans, magnetic resonance imaging (MRI), and X-rays, to detect abnormalities or potential cancerous lesions. They aid in early diagnosis and increase the chances of successful treatment against ES [24,25].

#### 2.1.2. Tumor Detection and Segmentation

Radiomic analysis uses algorithms to analyze image data to extract beyond what is visually apparent. The tumor segmentation step is the most crucial since it converts the original medical image into an extractable image. Despite prolonged studies, there is still room for improvement in the fully automatic segmentation technique, especially in medical image analysis [26]. AI algorithms can assist the comprehension of MRI, CT, and ultrasound images to precisely identify and segment the tumors, even in pediatric patients when the tumors are much smaller, and the contrast is considerably lower. The most relevant methods for segmenting images are threshold segmentation, image segmentation methods based on fuzzy logic, region-based image segmentation, and edge-based image segmentation. Although there are a lot of algorithms in tumor image segmentation, the following issues are still not well resolved: the best segmentation algorithm for use in different scenarios, how different segmentation algorithms will affect post-feature quantification and feature extraction, which type of segmentation method will be more in line with the gold standard, and whether there exists a general segmentation method [27].

#### 2.1.3. Quantitative Analysis of Tumor Characteristics

Quantitative image analysis is used to extract quantifiable tumor characteristics from medical images [28]. These tumor imaging biomarkers can be extracted from medical images such as MRI or CT. AI enables the extraction of quantitative features from medical images, assessing tumor size, shape, texture, and enhancement patterns. These features aid in differentiating between benign and malignant lesions and monitoring tumor response to treatment [29,30].

Quantitative imaging and analysis of characteristics such as tumor shape, size, texture, heterogeneity, and molecular markers are important for the diagnosis, staging, prognosis, and evaluation of treatment response for Ewing Sarcoma. This is made possible by AI algorithms, including machine learning and deep learning, through the review of advanced, high-dimensional data from multimodality imaging and digital pathology slides to uncover patterns undetectable by inhuman vision [31].

Radiomics, a subfield of AI, automatically derives a rich quantitative feature set from medical images such as X-rays, MRI, CT, and PET scans [32]. In ES, radiomics models enable efficient discrimination between tumor and non-tumor tissues, the quantification of tumor heterogeneity, and the prediction of clinical status. For example, radiomics analysis of MRI imaging has been used to predict patient response to neoadjuvant chemotherapy and characterize tumor subtypes according to textural and morphological features [33]. Outside of radiology, AI software analyzes digital pathology images for the purpose of evaluating tumor morphology and subtypes with high accuracy. Recent advancements include machine learning algorithms trained on computerized hematoxylin and eosin-stained slides that can predict sarcoma subtypes, including Ewing Sarcoma, with over 90% accuracy [34].

#### 2.1.4. Risk Stratification and Prognostic Assessment

Risk stratification is mainly determined using tumor location, pathological tumor grading, tumor size, and specific histological subtypes [35,36]. AI models can integrate imaging data with clinical and molecular information to stratify patients into risk groups and predict outcomes such as overall survival, disease-free survival, and risk of recurrence. This information helps clinicians tailor treatment strategies to individual patients [37].

#### 2.1.5. Radiomics and Texture Analysis

Radiomics is an image-processing method that uses data extraction to identify biomarkers for customized treatment [38]. AI-driven radiomic analysis can identify subtle imaging biomarkers associated with treatment response and prognosis, aiding in personalized treatment planning. AI and radiomics can work together because AI can handle enormous amounts of data more effectively than standard statistical techniques. The main goal is to extract and analyze as many relevant hidden quantitative data as possible to support decision-making processes.

Texture-based MRI texture models have been shown to be very sensitive in distinguishing between histological osteosarcoma subtypes such as chondroblastic versus non-chondroblastic patterns via intraosseous and extraosseous lesion compartment assessment [39]. Models using T2-weighted images have been able to distinguish these patterns with AUCs of up to 0.89 during validation, highlighting the potential of texture features to delineate subtle tumor histology not easily distinguishable by means of conventional imaging [39]. Such models allow for early risk stratification to guide clinical management plans, including chemotherapy aggressiveness and timing of surgery. PET radiomics provides added richness to diagnostic accuracy through the inclusion of metabolic tumor features reflecting tumor microenvironment heterogeneity and metabolic activity, which are key predictors of tumor behavior in children with sarcomas. Radiomics is also useful in differentiating pulmonary metastases from non-metastatic nodules in pediatric osteosarcoma patients according to CT texture analysis, yielding higher diagnostic accuracy than conventional radiological assessment [40].

#### 2.1.6. Clinical Decision Support Systems (CDSS)

A computer system that gives physicians information and tools to aid decision-making is called a clinical decision support system (CDSS) [41]. There are two types of CDSSs: knowledge-based and non-knowledge-based. Rules, like IF-THEN statements, are used by knowledge-based systems to analyze inputs and generate outputs or actions. Rules can be based on literature, practice, or patient-directed evidence [42]. The CDSSs can be classified into Expert System (ES)-based and Machine Learning (ML)-based systems. The ES-based CDSS is relatively simple, and evaluations are based on binary decisions. On the other hand, ML-based CDSS is complex and based on statistical inferences [43]. AI-powered CDSSs assist radiologists and oncologists in interpreting imaging findings, providing risk estimates, and recommending optimal treatment strategies based on personalized patient data [44].

### 2.2. AI Image Analysis Tools and Algorithms in Pediatrics Cancers

Several AI image analysis tools and algorithms are being developed and used in pediatric oncology to assist in detecting, diagnosing, and treating cancers. They include the following categories:

#### 2.2.1. Convolutional Neural Networks (CNNs)

A CNN is a kind of artificial neural network mainly used for processing and image recognition because of its capacity to identify image patterns [45]. CNNs are widely used in medical image analysis, including pediatric oncology. They can automatically learn hierarchical representations of features from medical images, enabling tasks such as tumor detection, segmentation, and classification [46]. Moreover, by integrating Grad-CAM explainability approaches, doctors may see regions of CT images that are mostly responsible for CNN-based predictions, increasing openness and confidence in AI-assisted diagnosis. In clinical settings, interpretability is crucial since decisions about invasive operations are mostly based on imaging results. To customize surgical strategies and radiation therapy for the treatment of Ewing Sarcoma, CNNs can provide whole-image assessments by identifying tumor margins and internal heterogeneity features [47].

#### 2.2.2. U-Net

U-Net is a convolutional neural network designed for biomedical image segmentation, modified for fewer training images and better precision [48]. The U-Net architecture has also been employed in diffusion models for iterative image denoising. U-Net is one class of popular CNN architectures dealing with segmentations of medical images, including pediatric oncology. It performs exceptionally well in segmenting tumors and organs from medical images against smaller datasets [49]. Recent research has used U-Net design modifications, such as nnU-Net, to accurately segment pelvic and sacral ES lesions. These models replace the laborious and arbitrary manual segmentation that radiologists have historically performed by automating the delineation of tumor areas. For staging and treatment decision making, U-Net models facilitate quicker diagnosis and more reliable tumor volume assessment by drastically lowering the need for manual annotation [47].

#### 2.2.3. DeepLab

DeepLab represents semantic segmentation architecture: first, the input image passes through the network by dilated convolution, while later, the output is processed by bilinear interpolation, and then a fully connected Conditional Random Field is applied to perform fine-tuning to obtain the final prediction [50]. DeepLab is a state-of-the-art deep learning algorithm for semantic image segmentation [50,51]. DeepLab has been applied in pediatric oncology for accurate tumor segmentation from medical images, aiding in treatment planning and monitoring [52]. The DeepLab model, especially the DeepLabV3 and DeepLabV3+ versions, has been shown to be highly efficient in semantic segmentation by utilizing atrous (dilated) convolutions and the Atrous Spatial Pyramid Pooling (ASPP) module, which capture multi-scale contextual information without a loss of spatial resolution. This functionality enables DeepLab to discriminate between tumor tissues and surrounding healthy tissues clearly, even if tumors present with complex shapes or at varied scales, a case often encountered in Ewing Sarcoma imaging [53]. The recent AI applications for pediatric cancers are shown in Table 1 [9].

## 3. AI Approaches in Drug Discovery and Development

### 3.1. Target Identification for Pediatric Cancers

AI is used to examine large datasets and find possible molecular targets for cancer therapy. Because pediatric malignancies are rare diseases that require specialized therapies for young children, drug research and development for these conditions present unique hurdles. AI-based technologies are being used more frequently to expedite the procedure and handle these issues [54,55].

### 3.2. AI in Anticancer Drug Repurposing

AI systems can examine enormous databases of currently available medications, genetic information, and disease characteristics to find possible candidates for repurposing in pediatric malignancies [56,57,58,59]. AI expedites the identification of novel treatment options by forecasting which medications may be effective against various kinds of cancer. The efficacy of existing anticancer medicines has been limited to a few tumor types despite enormous efforts in academic and pharmaceutical research worldwide [60]. The acknowledged obstacles to creating new molecular entities are that the novel drug design is more costly, slower, less safe, and more complicated than drug repurposing [61]. The extensive uses of machine learning algorithms and computational modeling have provided significant insights into the biological mechanisms of cancer and drug action [62].

### 3.3. AI in Anti-Drug Repositioning Based on Drug–Target Interaction

Numerous artificial intelligence-based methods have been used to predict the relationships between drugs and their targets [63]. Predicting drug–target connections is currently one of the primary methods for repurposing drugs [64]. Cheng et al. have developed a genome-wide localization system network algorithm to enable personalized medicine repurposing utilizing genomic data [65]. This technique obtains disease modules for drug repurposing using patient-specific DNA and RNA sequencing profiles of specific targets [65]. DeepDRK is a deep learning framework that was proposed by Wang et al. using kernel-based data integration. More than 20,000 pairs of anticancer medication combinations from pan-cancer cell lines were used to train the algorithm, utilizing kernel-based similarity matrices that incorporate data from multiple sources and fields such as genomics, transcriptomics, epigenomics, chemical characteristics of substances, and established drug–target interactions. By combining pharmacogenomic data, they offered a computational method for predicting the responses of cancer cells to medications, providing an alternate strategy for drug repurposing in cancer precision medicine [66].

### 3.4. AI in Anti-Drug Repositioning Considering Drug–Disease Interactions

Similarity and network analyses are the primary methods for identifying drug–disease interactions. A multiscale drug–disease topology learning framework (MTRD) using similarity-based techniques has been proposed [67]. This approach examined the novel therapeutic impact of existing pharmaceuticals by learning the representative characteristics of the drug–disease node pairings based on the relevant similarity and association data [68]. Similarly, to forecast new drug–disease interactions using drug-related and disease-related similarity information and previous drug–disease interactions, Jarada et al. introduced a unique deep learning-based framework called SNF-NN [69].

A new computational technique called MBiRW was proposed by Luo et al. to find possible new indications for well-known medications. This method combines similarity measures with the birandom walk (BiRW) algorithm [70]. Additionally, Sadeghi et al. presented a novel model for drug repositioning using multiple labeling of heterogeneous graph neural networks called DR-HGNN [71]. A graph neural network-based drug repositioning model known as GDRnet proposed by Doshi et al. showed the ability to effectively search the database for currently available medications and forecast their unidentified therapeutic effects [72].

### 3.5. Virtual Screening and Molecular Docking

A computer method called virtual screening (VS) is used in drug discovery to find possible therapeutic candidates from vast libraries of chemical compounds. VS is a fundamental method in contemporary drug discovery that is increasingly being employed to find new medicines against cutting-edge therapeutic targets [73,74]. Molecular docking algorithms are helpful in the identification of new therapeutic candidates because they can predict the interactions and binding affinities between medicines and target proteins [75,76,77,78].

### 3.6. AI’s Role in Evaluating Drugs for Cancer Prevention

Proteins or enzymes are the therapeutic targets used in the screening process for anticancer pharmaceutical hit chemicals. High-throughput screening is used primarily to find computer-aided hit chemicals by structure- and ligand-based screening [73]. In numerous research and development initiatives, high-throughput screening methods have proven to be quite effective; however, the efficiency of screening millions of compounds has reached a limit, and the expense is also substantial. The increasing use of GPUs, growing computing power, and the rapid advancement of AI technology have led to the development of more virtual hit compound screening techniques to broaden the toolkit for drug discovery [79,80].

#### 3.6.1. AI and Structure-Based Virtual Screening (SBVS)

The SBVS, which uses docking and scoring values against the target protein, is a significant method for identifying lead chemical compounds. Anticancer drug design benefits greatly from this tactic; however, many existing docking techniques are laborious and create barriers to extensive virtual screening [81,82]. The Similarity of Interaction Energy Vector Score (SIEVE-Score) is a new SBVS technique for hit compounds using AI approaches, which offers significant advantages over previous cutting-edge virtual screening techniques [83].

#### 3.6.2. AI and Ligand-Based Virtual Screening (LBVS)

LBVS is another screening method promptly used in drug discovery to identify similar chemical scaffolds from large datasets based on chemical and physical properties [84,85]. The recommended methods used for LBVS are the fingerprint approach [86], Shape-based similarity [87,88], and Pharmacophore modeling [89]. Moreover, there are some algorithms used in LBVS, such as the Tanimoto coefficient (a standard metric used to measure the similarity between fingerprints), Dice coefficient (similarity measure used for binary fingerprints), Shape overlay algorithms (3D shapes of molecules to identify the best spatial fit), and ML algorithms, being trained on known ligand data to learn how to identify new ligands with desired properties [90].

#### 3.6.3. AI and Fragment-Based Virtual Screening (FBVS)

Another AI-based computational drug discovery method called FBVS screens libraries of short molecule fragments to find possible candidates for drugs [91]. The goal of FBVS is to target relevant macromolecular targets with fragments of low molecular weight. Typically, FBVS generates potential drugs using chemical fragments with low molecular weight, low binding affinity, and simple chemical structures [73]. The application of nuclear magnetic resonance (NMR) for FBVS has gained popularity throughout the past 20 years. Faster medication development and lower production costs have been made possible by FVBS, resulting in a high success rate [92]. Several FBVS techniques are in use today, and ligand generation from a fragment through machine learning has become more popular. The primary benefit of utilizing FBVS is the low complexity of the simulation’s pieces, which permits the application of various methodologies to create novel compounds and mitigate drug development expenses [93].

### 3.7. AI-Based Target Identification for Anticancer Drugs

The first stage in designing an anticancer therapy is to identify drug–target interactions (DTIs). Binding affinity constants, which include markers like a dissociation constant (Kd), an inhibition constant (Ki), and a half-maximum inhibitory concentration (IC_50_), are frequently used to characterize the strength of drug–target binding. The computational prediction of DTIs is quite interesting because experimentally determining them takes a long time and a lot of money. Precise and efficient DTI forecasts can significantly support medication discovery and hasten the identification of lead or hit compounds [54,94].

Molecular docking simulation and machine learning-based techniques have historically been used as computational approaches for DTI predictions [95]. However, conducting these investigations without knowledge of the 3D structures of the pharmacological targets would be costly, time-consuming, and challenging. An innovative end-to-end learning system based on heterogeneous graph convolutional networks (EEG)-DTI is used to forecast DTI [96]. Drug and target low-dimensional feature representations were learned using a graph convolutional network-based model, which was then utilized to predict the DTI. Even without the utilization of the 3D structures of the pharmacological targets, it produced a promising DTI prediction performance. Shao et al. enhanced the prediction performance by treating the DTI prediction as a link prediction problem [97]. Yang et al. introduced a drug–target interaction prediction technique that relies on mutual learning mechanisms, even without 3D structure information, and tackles the deep learning explanation problem [98].

### 3.8. Determining the Druggability of Cancer Drug Targets Using AI

In designing drugs for cancer, choosing therapeutic targets is particularly crucial because it dramatically affects the likelihood that subsequent clinical trials will be successful [54]. As a result, numerous related techniques were created. Raies et al. presented a prediction model known as DrugnomeAI to solve the issue of tailored drug synthesis. DrugnomeAI was developed to forecast the druggability of drug targets in the human exome using a stochastic semi-supervised machine learning framework. Additionally, DrugnomeAI can be used to predict a drug target’s druggability in cancer disorders [99]. Synthetic lethality (SL) has recently been shown in many research papers to be a viable strategy for finding targets for anticancer medications. However, there are issues with the wet experimental screening for SL, such as excessive expenses, batch effects, and results that are not on goal [100]. Wang and colleagues developed KG4SL, a new model built on top of a graph neural network (GNN). It integrates a graph neural network prediction with knowledge graph (KG) messaging. The experimental findings showed that adding KG to the GNN for SL predictions had a significant positive impact [101].

### 3.9. Modelling Applications in Drug Discovery

#### 3.9.1. Variational Auto-Encoder (VAE) Model

Diederik P. Kingma and Max Welling introduced the variational auto-encoder (VAE) in 2013, which is a significant generative model [102]. To produce candidate compounds with anticancer therapeutic qualities, Born et al. used a hybrid VAE model, and the model produced strong inhibitory effects against particular diseases. In terms of structure, synthesizability, and solubility, the produced compounds resembled those of currently available medications [103]. The NEVAE model proposed by Samanta et al. addressed the issues with the existing approaches. For example, current models can only produce molecules with an equal number of atoms but do not use a huge number of macromolecules during training to limit the diversity of the molecules formed. They also do not provide the spatial coordinates of the produced atoms [104].

#### 3.9.2. Recurrent Neural Network (RNN) Model

The recurrent neural network (RNN) model creates molecules in a temporal order using fundamental units as the core vocabulary, such as atoms or molecules in fragments. The RNN model’s next atom character’s output probability is determined by the atom that came before it. A novel bidirectional RNN molecule generation model called BIMODAL was proposed by Grisoni et al. and is helpful for data augmentation and using Simplified Molecular Input Line Entry System (SMILES) strings. By using alternate Learning, the model accomplished bidirectional molecular design. It was then compared to other bidirectional RNNs. BIMODAL outperformed state-of-the-art techniques and showed promise in terms of molecular innovation, backbone diversity, and chemical and biological significance of the produced compounds [105].

#### 3.9.3. Generative Adversarial Network (GAN)

The generative adversarial network (GAN) is an unsupervised learning method comprising two networks: the generating network G, which matches the data distribution, and the discriminative network D, which assesses if the input is “real.” During the training process, the two networks play a game in which the generative network D attempts to extract as much real data as possible from the generative network’s output, while the generative network G attempts to “cheat” D by accepting random noise to mimic the real images in the training set. Ideally, the game should generate a “faked” generative model [106]. The Mol-Cycle GAN approach was proposed by Maziarka et al. [68,107]. Mol-Cycle GAN is a generative model-based conditional generative adversarial network technique for de novo drug design and optimization of molecule synthesis. Given an initial molecule, it can resolve the issue of molecules that are difficult to synthesize.

Additionally, it can produce molecules with the desired architectures and characteristics [68,107]. ABbbasi et al. [108] proposed a feedback-based GAN framework that connected a predictor depth model, a GAN, and an encoder–decoder via a feedback loop to apply an optimization method. The outcomes demonstrated that the GAN optimization model can produce compounds with high binding affinities [108].

### 3.10. Practical Application of AI to Treat Pediatric Cancer

The implementation of AI in pediatric oncology is especially challenging due to the rarity and heterogeneity of pediatric cancers and the paucity of large, standardized datasets [109]. Nonetheless, AI-powered precision medicine platforms are beginning to show substantial benefits in guiding the treatment of pediatric cancers. There are different applications of AI in pediatric cancer treatment [110]. AI algorithms, particularly deep convolutional neural networks (CNNs), can read medical images (MRI, CT, and PET scans) fast and accurately to detect very small anomalies indicative of cancer, which could lead to earlier diagnosis [111]. Furthermore, Computer-Aided Diagnosis (CAD)-based AI applications can also be utilized to assist pathologists in identifying cancer cells and tumor subtype classification from biopsies and pathology slides, improving diagnostic accuracy and speed [112]. AI has the capability to analyze genomic, proteomic, and metabolomic data in huge amounts to identify new biomarkers that will be used in the early detection of cancer and risk stratification in children [113]. AI models can integrate clinical, genomic, and imaging data to predict the response of a patient to a range of treatments, allowing oncologists to personalize therapies for best efficacy and minimum side effects [114]. AI can be used to precisely define tumors in order to plan radiation therapy, which could minimize radiation to normal tissues and optimize the outcome [115].

## 4. Recent Advancements in Pediatric Oncology Diagnosis and Treatment

ML is a subset of AI that deals with the development of algorithms capable of learning from data and predicting outcomes or making decisions without explicit programming [116]. ML algorithms can analyze complex datasets, identify patterns, and create predictive models. There are two main types: supervised learning with labeled data and unsupervised learning with unlabeled data, both used in healthcare. Deep learning, which uses deep neural networks, excels in processing unstructured data like medical images and genomic sequences [9]. ML and DL applications in pediatric oncology encompass several important aspects including diagnosis, tumor classification, treatment response prediction, and survival prognosis. Both technologies exploit different kinds of data including clinical records, pathology images, radiological imaging, and multi-omics datasets to allow comprehensive and integrative analyses [117,118]. In diagnostic applications, ML and DL models significantly enhance the accuracy of pediatric hematological malignancy classification [117]. For instance, DL on bone marrow cell microscopy images enables the classification of acute lymphoblastic leukemia (ALL), acute myeloid leukemia (AML), and chronic myeloid leukemia (CML) with accuracy exceeding 90%, even outperforming experienced hematologists in some instances [119]. Hybrid models combining genetic algorithms and residual CNNs have also optimized the diagnoses with accuracy rates of over 98%. Similarly, ML-based analysis of DNA methylation and transcriptomic data provides robust biomarkers allowing the determination of leukemia subtypes, which is crucial for individualized treatment planning. Furthermore, AI-driven intraoperative diagnostics, including Raman spectroscopy with ML classifiers, facilitate real-time tumor recognition and surgical guidance [9].

Research on extracranial tumors in children also benefits from AI techniques. From radiography and histopathologic data, ML classifiers can accurately differentiate between malignancies in bone and soft tissue tumors, improving diagnosis, particularly in environments with limited resources. In addition to reducing intrusive procedures, AI and ML technologies used to circulate tumor DNA and DNA methylation analysis are assisting efforts for liquid biopsies and early detection [120,121,122]. Furthermore, ML and DL enable prognostic modeling and therapeutic decision making [123]. Predictive models from electronic health records and imaging forecast treatment response, relapse risk, and cognitive function following therapy in children with leukemia, paving the way for risk-adapted trials and precision medicine. The models incorporate clinical, genomic, and imaging variables, enabling stratification to inform the intensification or de-escalation of therapies [123,124]. Many AI-based algorithms, such as deep learning, have emerged as effective instruments for AI-assisted anticancer medication development. A comparison of different models with accuracy and parameters is depicted in Table 2.

## 5. Analysis of Genomic and Molecular Data

AI is also applied when examining pediatric cancer patients’ transcriptome, proteome, and genetic data to find promising molecular targets for future medication development. AI contributes to developing targeted medicines suited to the unique molecular profiles of pediatric patients by identifying genetic changes that fuel cancer growth.

### 5.1. Pediatrics Malignancies and Genome Landscape

Early large-scale sequencing studies of pediatric malignancies highlighted the generally low mutational burden while identifying novel driver genes [125]. Furthermore, the whole-genome sequencing (WGS) investigations of acute myeloid leukemia (AML), T cell acute lymphoblastic leukemia (T-ALL), and Wilms tumor revealed subtype-specific driving events and highlighted the interaction between germline and somatic changes in the pathogenesis of juvenile cancer. These investigations have identified several pathways unique to juvenile cancer [125,126]. For instance, pediatric AML is distinguished by frequent age-dependent gene fusion events and focused regions of gene deletion; however, both adult and pediatric AML are generally characterized by a low mutational burden and a lengthy “tail” of uncommon mutational events [127]. Furthermore, it was shown that certain co-occurring occurrences, such as a NUP98-NSD1 fusion or FLT3-ITD (internal tandem duplication) with the WT1 mutation, were indicative of poor outcomes, particularly in pediatric AML [128].

### 5.2. AI Resources for Genome Landscape Research

Analyses of the enormous and complex amounts of data produced by pediatric cancer genome studies have been completely transformed by AI [129]. The essential tools and resources that play a significant role in understanding pediatric cancer through genomic studies are listed in Table 3.

## 6. Conclusions

Cancer diagnosis has been transformed with the advent of AI methods. AI techniques have been applied broadly in adult cancers and pediatrics. However, there are very few specialized uses of AI algorithms in children’s cancer, most likely because there are not sufficient datasets to understand the etiology of many pediatric cancers better. Most CNNs cannot be simply generalized from children to adults, and thus well-trained CNN architectures learned from adults cannot be applied directly to pediatric oncology. Therefore, AI algorithms specifically developed for use in pediatric oncology are required. Based on the gathered data, there is immense scope for application in pediatric cancers using AI, which can be validated for application in assisting therapeutic decisions in the near term.

Despite promising results, the application of ML and DL in pediatric oncology is confronted with certain field-specific limitations. Foremost among them is the unavailability of large, high-quality pediatric datasets due to the low incidence of pediatric cancers and ethical limitations in pediatric research. Small sample sizes limit the ability of ML models, especially data-hungry deep learning architectures, to generalize to diverse pediatric populations [139]. The heterogeneity of childhood tumors also hinders the development of models, as clinical and molecular diversity require integrative, multi-modal datasets that are rarely available in adequate quantities. The standardization and interoperability of data across centers are still lacking, limiting collaborative studies and external validation [140]. Privacy and ethical issues are significant hurdles in data sharing in pediatrics, where stringent protections sometimes restrict data availability for model training. Moreover, the interpretability of complex ML/DL models is crucial in the clinical environment to establish trust among clinicians; reliance on “black-box” deep learning methods resists transparency and accountability of decisions [141].

Regulatory frameworks governing the application of AI in medicine are being developed continuously, generating uncertainties regarding model validation, approval, and continuous monitoring, especially for pediatrics-specific tools. Practical challenges include compatibility with existing clinical workflow and electronic health records, computational costs, and differences in technical infrastructure across sites of care. The subject of machine learning in pediatric oncology is still in its infancy, according to this systematic study. The field will advance with the help of enhanced methodology, larger datasets, and uniform reporting standards. Numerous potentials exist to use machine learning techniques in pathology, imaging, and electronic health records to find novel biomarkers, algorithms, and tools to enhance the care of children with cancer.

Advancements in transfer learning, where the model is pre-trained on adult oncology data and subsequently fine-tuned for pediatric use, promise to alleviate data constraints. Federated learning permits simultaneous model training without data exchange, ensuring privacy while improving robustness. The further incorporation of ML with multi-omics and multi-modal imaging databases promises to unlock new biomarkers and therapeutic directions [140]. The development of XAI techniques is important to enhance the transparency and acceptability of ML models in pediatric oncology to enable clinicians to understand model thinking and integrate predictions into clinical decision-making [139]. The standardization of data collection protocols, expansion of open-access pediatric oncology databases, and encouragement of interdisciplinary collaborations will be the building blocks of future progress. Furthermore, integrating ML and DL into clinical trials and treatment regimens could facilitate adaptive, precision pediatric oncology treatment, enhancing survival and limiting long-term toxicity. Novel AI applications could support pharmacogenomics, fine-tune radiotherapy doses, and assist in the identification of patients at risk for treatment toxicities.

## Figures and Tables

**Table 1:** Current AI applications in the diagnosis of childhood non-solid tumors.

AI Method	Medical Field	Task	Tumor
CNN/GAN	Pathology	Detecting ALL and AML using a deep learner classifier using microscopic blood images	ALL and AML
CNN and GAN	Pathology/Genomics	Constructing a hybrid model using a genetic algorithm and a residual CNN to predict ALL using microscopy images	ALL
SVM	Pathology	Building a model to classify acute leukemias using flow cytometry	Acute promyelocytic leukemia
ANN/FFNN/SVM	Pathology	Proposing an ML-based model for ALL categorization using microscopic blood images	ALL
CNN	Pathology	Building an aggregated DL model for leukemic B-lymphoblast classification	Leukemic B-lymphoblast
CNN	Pathology	Using bone marrow cell microscopy images for the classification of AML, ALL, and CML	AML, ALL, and CML
RF	Others-mRNA sequencing	Developing transcriptome-wide biomarkers for ALL subtyping	ALL
ANN	Others-DNA methylation	Identifying reliable cancer-associated methylation signals in gene regions from leukemia patients	Leukemia
Nearest shrunken centroids	Others-DNA methylation	Investigating the utility of CpG methylation status to differentiate blood from patients with ALL and AML from normal blood	ALL and AML

GAN: generative adversarial network; SVM: support vector machine; ANN: artificial neural network; FFNN: feed forward neural network; ACC: accuracy; AUC: area under the curve; RF: random forest; CpG: cytosine-guanine.

**Table 2:** Comparison of AI algorithms along with different key features.

AI Models	Cancer Type	Data Type	Application	Performance Metrics	Key Features
Deep Learning CNN	Acute Lymphoblastic Leukemia (ALL), AML, CML	Bone marrow cell microscopy images	Classification of leukemias	Accuracy: 90–99%; Some models >98%	Outperforms expert hematologists; hybrid models improve with genetic algorithms
Transcriptomic & Methylation-Based ML	ALL Subtyping	mRNA sequencing, DNA methylation profiles	Subtyping and diagnosis	Accuracy: 93.8–100%; AUC up to 99.98	Uses multi-omics; provides reliable leukemia subtype discrimination
ML Classification (SVM, RF)	Acute Promyelocytic Leukemia	Flow cytometry	Leukemia classification	ACC: 94.2%; AUC: 99.5	Effective in flow cytometry data
DL-CNN	Pediatric Brain Tumors (Medulloblastoma, Gliomas, Ependymomas)	MRI sequences, Histological images	Tumor subtype classification and diagnosis	Accuracy: 75–95.5%; AUC: 0.81–0.99	Improved sensitivity and specificity; supports real-time and intraoperative diagnostics
Temporal Deep Learning Model	Pediatric Gliomas	Sequential brain MRI scans	Predict recurrence risk	Accuracy: 75–89%	Uses temporal learning on multiple longitudinal scans, outperforming single-timepoint methods
DL-Based PET/MR Image Augmentation	Pediatric Lymphoma	Ultralow-dose PET/MR images	Image quality enhancement, dose reduction	Radiation dose reduction >90%	Augments image quality to reduce radiation exposure in imaging
ML Radiomics + CT	Osteosarcoma	CT scan of primary tumor	Predict lung metastases	Accuracy: 73%	Early metastatic risk prediction; needs further validation
DL-CNN + Multimodal MRI	Osteosarcoma	MRI images (T1, STIR, postcontrast)	Chemotherapy response evaluation	Accuracy: >90%	Differentiates necrotic vs. viable tumor areas
ML Model Using FDG PET	Osteosarcoma	Baseline FDG PET imaging	Predict neoadjuvant chemotherapy response	AUC: Up to 0.863	Texture feature analysis improved response prediction
CNN Classifier	Ewing Sarcoma	Radiographs	Lesion detection and differentiation	Accuracy: ~90–94%	Differentiates Ewing sarcoma and osteomyelitis effectively
DL-CNN Classifier	Wilms Tumor	Triphasic CT images	Tumor differentiation and staging	Sensitivity: 78.1%; Accuracy: ~79%	Outperforms human experts for non-Wilms tumor detection
ML Classifiers (SVM, RF etc.)	Soft-Tissue Sarcomas	Radiological images, histopathology slides	Malignant vs. benign differentiation	Accuracy: 80.8–90.5%; AUC: 0.88–0.96	Applied on histopathology and imaging, effective in pediatric soft-tissue masses classification
CNN-Based Dermatology AI	Infantile hemangiomas	Clinical and dermoscopic photos	Disease diagnosis	Accuracy: 91.7%	Non-invasive clinical image-based AI diagnosis
ML & Proteomics	Pediatric Brain Tumors	CSF proteomic profiles	Tumor subtype classification	AUC: 0.97–1	Classifies brain tumors accurately with proteomics and ML algorithms
Raman Spectroscopy + ML	Intraoperative Pediatric Brain Tumors	Raman spectroscopy data	Real-time tumor vs. normal tissue differentiation	AUC: 0.91–0.94	Enables safe tumor resections intraoperatively

**Table 3:** AI-based implication models for pediatric oncology.

AI Approaches	Explanation	Ref.
Variant identification	Identify and classify the somatic and other genomic alterations from sequencing data.	[130]
Pattern recognition and discovery	Discover hidden relationships and patterns in large datasets to identify new driver genes, pathways, and possible treatment targets.	[9,131]
Integration of multi-omics data	Integrate information from genomics, transcriptomics, proteomics, and other omics data sources for treatment prediction.	[132,133]
Predictive modeling	Make informed clinical decisions and enhance the care of individual patients by using their genetic profiles to predict treatment success, relapse risk, and patient outcomes.	[134]
Clinical Trial Design and Patient Stratification	Optimize clinical trial design and predict treatment responses based on patient characteristics and molecular profiles.	[135]
Prognostic and Predictive Analytics	Survival prediction and recurrence risk for the patients	[136]
Telemedicine and Remote Monitoring	Remote consultations and continuous monitoring of patients	[137,138]
